# COVID-19: Multiorgan Dissemination of SARS-CoV-2 Is Driven by Pulmonary Factors

**DOI:** 10.3390/v14010039

**Published:** 2021-12-26

**Authors:** Akmaljon Odilov, Alexey Volkov, Adhamjon Abdullaev, Tatiana Gasanova, Tatiana Lipina, Igor Babichenko

**Affiliations:** 1 Department of Pathological Anatomy, Peoples′ Friendship University of Russia (RUDN University), 6 Miklukho-Maklaya St, Moscow 117198, Russia; alex.volkoff@gmail.com (A.V.); babichenko@list.ru (I.B.); 2 Department of Pathological Anatomy, Municipal Clinical Hospital Named after E.O. Mukhin, Moscow 111399, Russia; 3 Laboratory of Molecular Hematology, National Research Center for Hematology, Novy Zykovski lane 4a, Moscow 125167, Russia; adham_abdullaev@mail.ru; 4 Department of Virology, Lomonosov Moscow State University, Leninskie gori, 1, 40, Moscow 119234, Russia; tv.gasanova@gmail.com; 5 Department of Cell Biology and Histology, Faculty of Biology, Lomonosov Moscow State University, Leninskie gori, 1, 12, Moscow 119234, Russia; tlipina@mail.ru

**Keywords:** COVID-19, SARS-CoV-2, multiplex real-time polymerase chain reaction, viral load, FFPE, multi-organ dissemination

## Abstract

Multi-organ failure is one of the common causes of fatal outcome in COVID-19 patients. However, the pathogenetic association of the SARS-CoV-2 viral load (VL) level with fatal dysfunctions of the lungs, liver, kidneys, heart, spleen and brain, as well as with the risk of death in COVID-19 patients remains poorly understood. SARS-CoV-2 VL in the lungs, heart, liver, kidneys, brain, spleen and lymph nodes have been measured by RT qPCR using the following formula: N^SARS-CoV−2^/N^ABL1 ×^ 100. Dissemination of SARS-CoV-2 in 30.5% of cases was mono-organ, and in 63.9% of cases, it was multi-organ. The average SARS-CoV-2 VL in the exudative phase of diffuse alveolar damage (DAD) was 60 times higher than in the proliferative phase. The SARS-CoV-2 VL in the lungs ranged from 0 to 250,281 copies. The “pulmonary factors” of SARS-CoV-2 multi-organ dissemination are the high level of SARS-CoV-2 VL (≥4909) and the exudative phase of DAD. The frequency of SARS-CoV-2 dissemination to lymph nodes was 86.9%, heart–56.5%, spleen–52.2%, liver–47.8%, kidney–26%, and brain–13%. We found no link between the SARS-CoV-2 VL level in the liver, kidneys, and heart and the serum level of CPK, LDH, ALP, ALT, AST and Cr of COVID-19 patients. Isolated detection of SARS-CoV-2 RNA in the myocardium of COVID-19 patients who died from heart failure is possible. The pathogenesis of COVID-19-associated multi-organ failure requires further research in a larger cohort of patients.

## 1. Introduction

COVID-19, as a multisystemic pathology, has led to more than 5 million deaths worldwide to date [1]. A severe course and fatal outcomes of the disease are commonly associated with the severe acute respiratory syndrome (ARDS) and multi-organ failure [2,3,4,5]. The end-organ failure in COVID-19 patients is generally represented by hepatic lesions in 14–29% of cases, renal injury in 19–29%, heart failure in 33% [6,7], cerebral lesions in 13–69% [8,9], and spleen infarctions in rare cases [10,11].

The pathogenesis of multi-organ failure in COVID-19 patients may be associated with cytokine storm [2,12], activation of the adaptive immune system [2], dysregulation of the renin–angiotensin–aldosterone system (RAAS) as a result of virus-mediated suppression of ACE2 expression [13], damage to endothelial cells and vascular inflammation [14], and sepsis [15]. Moreover, the multi-organ tropism of SARS-CoV-2 is considered one of the potential mechanisms of multiple organ failure [16,17].

According to the literature, elevated levels of serum creatine phosphokinase (CPK), aspartate aminotransferase (AST), alanine aminotransferase (ALT), lactate dehydrogenase (LDH), alkaline phosphatase (ALP), and creatinine (Cr) might be used as indicators of COVID-19 associated heart, liver and kidney injury [18,19,20,21,22]. For instance, acute myocardial injury is associated with a rise in serum CPK and troponin [19], liver injury (with high levels of aminotransferases) [20], and kidney injury (with elevated Cr level) [21]. In addition, a severe course and fatal outcomes of COVID-19 due to multi-organ injury are associated with high LDH [22].

SARS-CoV-2 RNA can be detected in the heart [23,24,25], liver [23,25,26], spleen [25,27], lymph nodes [25,27], brain [25,27,28] and other tissue samples [25,29,30] via real-time polymerase chain reaction (RT PCR). Immunohistochemical (IHC) examination revealed SARS-CoV-2 antigens in the lungs [31,32,33,34], liver [35,36], kidneys [25,35,37], heart [35], brain [28] and lymph nodes [33,38,39,40]. In addition, SARS-CoV-2 virions were visualized using transmission electron microscopy (TEM) in pneumocytes, alveolar macrophages and endothelial cells of the lungs [41,42,43], cardiomyocytes and interstitial cells of the heart [44,45], epithelial cells of the proximal tubules of the kidneys [46] and liver histiocytes [20,36].

Most of the research, as mentioned earlier, is just limited to the identification of SARS-CoV-2 infection in tissues without quantifying the viral load (VL) or to an assessment of the SARS-CoV-2 VL relative to the threshold cycle (Ct) of RT PCR [27,31]. However, the SARS-CoV-2 VL level expressed in the number of Ct of RT PCR should be treated very carefully, since these values strongly depend on the quality and amount of the extracted RNA, the efficiency of primers and probes, and the passage of the amplification reaction [47,48]. Therefore, the College of American Pathologists urges caution in interpreting the cycle threshold (Ct) values when assessing the SARS-CoV-2 VL in COVID-19 patients [49].

Based on this, we developed an original method for quantitative RT PCR, which allows us to calculate the actual copies of the SARS-CoV-2 cDNA and the human *ABL1* gene relative to the standard curve constructed based on serial dilutions (with a correlation of R2 ≥ 0.99) of the plasmid vector pGEM^®^- T Easy containing inserts of the SARS-CoV-2 genome and the *ABL1* gene [50].

Currently, several questions regarding the “pulmonary mechanisms” of multiorgan spread, dissemination patterns, and the SARS-CoV-2 VL level in the vital organ tissues of patients who died from COVID-19 remain open. In addition, it is also necessary to assess the pathogenetic contribution of SARS-CoV-2 infection and viral load to developing multiorgan dysfunction in COVID-19.

We believe that a study of the VL level of SARS-CoV-2 and the nature of morphological changes in the “primary focus” of inflammation (in the lungs) will reveal possible “pulmonary factors” that contribute to the extrapulmonary spread of SARS-CoV-2. Thus, investigation of the dissemination frequency, the level of SARS-CoV-2 VL in the tissues of the most frequently affected organs, and their relationship with biochemical markers of their functional activity will make it possible to assess the pathogenetic contribution of SARS-CoV-2 tissue infection to the development of multi-organ dysfunction in patients with COVID-19.

## 2. Materials and Methods

We used formalin-fixed and paraffin-embedded (FFPE) blocks of brain (*n* = 33), lung (*n* = 143), heart (*n* = 36), liver (*n* = 36), spleen (*n* = 35), kidneys (*n* = 36) and lymph node (*n* = 35) tissues, taken for histological studies during the autopsy of 36 patients who died of COVID-19. According to the ninth version of the Russian Interim Guidelines for the Prevention, Diagnosis, and Treatment of New Coronavirus Infection (COVID-19) dated 26 October 2020, only tissue samples fixed in 10% neutral buffered formalin solution for at least 24 h can be considered biologically safe. The study was approved by the local Medical Ethical Committee (protocol #22/2020) and carried out following the Declaration of Helsinki’s rules of 2013.

### 2.1. Quantitative RT PCR and SARS-CoV-2 VL Measuring Method in Tissues of Various Organs

RNA was isolated from 10–12 3-μm-thick FFPE tissue sections of brain, lung, heart, liver, spleen, kidneys, and lymph node tissue blocks using a kit of reagents for RNA isolation from FFPE tissues, PureLinkTM FFPE (Invitrogen Corporation, Carlsbad, CA, USA) and innuPREP FFPE total RNA Kit (AJ Innuscreen GmbH, Berlin, Germany), according to the manufacturer’s instructions. To obtain cDNA in a volume of 20 µL, a reverse transcription reaction was performed using a 10 µL RNA solution and a set of reagents “Reverta-L” (InterLabService Ltd., Moscow, Russia), following the manufacturer’s instructions. The quantitative multiplex RT PCR and the method for calculating the SARS-CoV-2 VL relative to the curves of serial dilutions of the plasmid vector pGEM^®^-Teasy (Promega Corporation, Madison, WI, USA) with the *ORF1ab* region of the SARS-CoV-2 cDNA and the human *ABL1* gene have been described previously [50].

### 2.2. Immunohistochemical and Transmission Electron Microscopic Detection of SARS-CoV-2

An immunohistochemical study was performed on 3-μm-thick FFPE tissue sections on positively charged glass slides. The detection of the coronaviral antigens was carried out using primary monoclonal antibodies against the spike (S) protein of SARS-CoV-2 (clone 1A9) produced by GeneTex, Inc. (Irvine, CA, USA) and the anti-SARS-CoV-2 spike glycoprotein antibody (ab272504) produced by Abcam plc (Cambridge, UK), by amplifying the signal through a multimeric detection system BenchMark ULTRA (Ventana Medical Systems, Inc., Tucson, AZ, USA). The transmission electron microscopic detection of SARS-CoV-2 in the tissues was performed according to the protocol described previously [50].

### 2.3. Statistical Analysis

For statistical analysis, we used the free and open statistical platform jamovi (version 1.6, https://www.jamovi.org, accessed on 10 July 2021).

## 3. Results

General characteristics of the 36 patients (21 men, 15 women) with a clinical diagnosis of COVID-19, the number of bed days spent in an intensive care unit (ICU) until fatal outcome, histopathology of lungs, the mean value of SARS-CoV-2 VL in the lungs, tracheobronchial lymph nodes, heart, spleen, liver, kidneys, and brain are presented in Table 1.

As demonstrated in Table 1, the frequency of SARS-CoV-2 RNA detection in the general cohort of patients, by organs, was: in the lungs–91.7% (33/36), tracheobronchial lymph nodes–58.8% (20/34), heart–40% (15/35), spleen–35.3% (12/34), liver–31.4% (11/35), kidneys–20.7% (6/29) and brain–9% (3/33).

All patients were divided into three groups according to a number of SARS-CoV-2 RNA-positive organs: patients in whom SARS-CoV-2 RNA was not detected in any organ sample–25.5% (2/36), patients with mono-organ–30.5% (11/36), and multi-organ–63.9% (23/36) dissemination.

The first group included two patients (a 62-year-old man and a 77-year-old woman) in whom, despite a lifetime clinical diagnosis of COVID-19, no SARS-CoV-2 RNA was detected in any of the tissues examined. The length of stay of patients in the ICU was 2 and 15 bed-days, respectively. Histopathological changes in the lungs of both patients corresponded to the proliferative phase of DAD, and patient №7 also had pulmonary emphysema.

The second group consisted of 11 patients (7 men and 4 women) aged 64–90 years, in whom SARS-CoV-2 RNA was detected only in one of the examined organs (mono-organ dissemination). In 10 of them, SARS-CoV-2 RNA was detected only in lung tissue samples and from patient No. 23 in cardiac tissue. The median ICU stay was 16 (range 9–24) days. Histological changes in the lungs in this group of patients corresponded to bronchopneumonia (*n* = 5) and the proliferative phase of DAD (*n* = 6). The median viral load in the lungs was 481 (27–1952) copies of SARS-CoV-2 DNA per 100 ABL1.

The third group included 22 patients (13 men and 9 women) aged 52–95 (median 77) years, in whom SARS-CoV-2 RNA was detected in two or more examined organs (multi-organ dissemination). In this group, the median ICU stay was 11 (range 1–27) days. Histological changes in the lungs in more than half of the patients, especially those with a viral load of ≥4909, were characterized exclusively by the exudative phase of DAD. The range of SARS-CoV-2 VL in the lungs of patients with multiorgan dissemination was 18–250,281 copies of SARS-CoV-2 cDNA per 100 ABL1. The SARS-CoV-2 VL range in the lymph nodes was 112–11,586, heart–270–6930, liver–7–9770, spleen–9–2899, kidneys–52–2899, and brain–718−2573 copies of SARS-CoV-2 cDNA per 100 copies of ABL1 cDNA.

Statistical analysis revealed a strong direct correlation between the histological phase of DAD with the SARS-CoV-2 VL level in the lungs (Spearman’s rho = 0.846, *p* < 0.001), and the frequency of SARS-CoV-2 dissemination in extrapulmonary organs (Spearman’s rho = 0.777, *p* < 0.001). Higher values of the VL level and a higher frequency of extrapulmonary dissemination were characteristic of the exudative phase of DAD. In addition, a negative correlation was found between the VL level in the lungs and the time spent in the ICU before death (rS = −0.348, *p* = 0.037). Patients with a shorter stay in the ICU had higher VL levels in the lungs, and vice versa.

**Figure 1:**
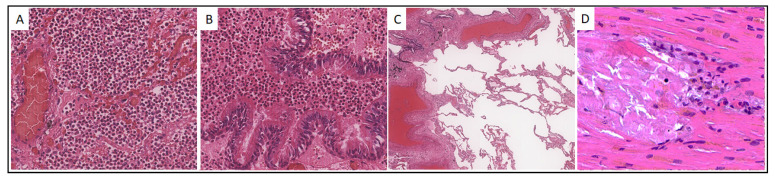
Histological picture of the lungs and myocardium of patient No. 23. (**A**,**B**). Intraalveolar and intrabronchiolar polymorphonuclear leukocyte infiltration, ×200; (**C**). Areas of peribronchiolar and perivascular fibrosis, and emphysematous enlargements, ×40; (**D**). Myocardial microinfarctions with mononuclear inflammatory infiltration, ×400. Hematoxylin-eosin staining.

Considering the uniqueness of the case of isolated SARS-CoV-2 RNA detection within the myocardium, the results of molecular genetic analysis for the detection of SARS-CoV-2 RNA in the tissues of the examined tissue samples of patient No. 23 is presented in Figure 1.

*Case 23.* A 90-year-old male was hospitalized on 30 October 2020 with intermittent body temperature up to 38 °C, cough, and low blood pressure equal to 70/50 mm Hg. However, a day later, due to a further blood pressure decrease to 30/80 mm Hg, the patient was transferred to the intensive care unit and for the next two days received norepinephrine infusion, first at a rate of 0.6 μg/kg/min, and then 1.0 μg/kg/min, up to death from cardiac arrest (3 November 2020). Histological examination of the lungs of Pt23 revealed features characteristic of bronchopneumonia with fibrotic changes and areas of emphysematous dilatation (Figure 1A–C). The histological picture of the myocardium was characterized by single foci of microinfarctions with mild mononuclear infiltration (Figure 1D).

**Figure 2:**
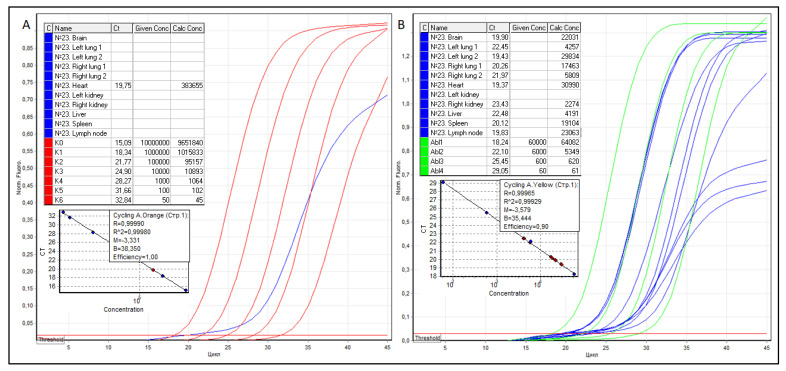
Threshold cycles (Ct), the concentration of serial dilutions of pGEM^®^-T Easy plasmids, the copy number of SARS-CoV-2 (**A**) and ABL1 (**B**) cDNA, as well as graphical RT qPCR curves of patient No. 23. The samples are colored blue, SARS-CoV-2 serial dilutions—red, ABL1 serial dilutions—green.

Further, from FFPE tissue blocks of the brain, both lungs, heart, kidneys, liver, spleen, and regional lymph node, sections were prepared, RNA was isolated, cDNA was obtained by reverse transcription, and RT qPCR was performed, the results of which are shown in Figure 2.

As shown in Figure 2, in the presence of 2274 to 30,990 copies of ABL1 cDNA in the studied samples, except for the myocardium, no SARS-CoV-2 RNA was detected anywhere. The true copy numbers of SARS-CoV-2 and ABL1 RNAs in the myocardium were 383,655 (R2 = 0.99980, efficiency–1.00) and 30,990 (R2 = 0.99929 and efficiency–0.90) copies, respectively. According to the formula N^SARS-CoV−2^/N^ABL1^ × 100, SARS-CoV-2 VL in the myocardium was 1238 copies of SARS-CoV-2 RNA per 100 copies of ABL1.

As an example of multiorgan dissemination of SARS-CoV-2, the results of histological examination and measurement of the VL level of Patient №10 are presented.

**Figure 3:**
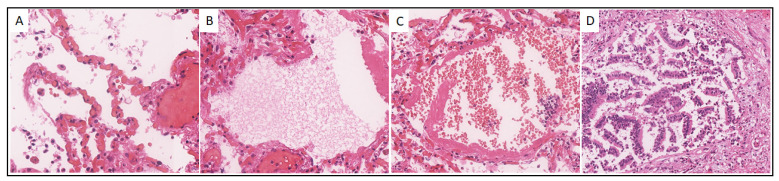
Histological picture of the lungs of patient No. 10. (**A**). Congestion and stasis of the capillaries of the interalveolar septa, intraalveolar cell detritus, ×300; (**B**)**.** Intraalveolar edema and hyaline membranes, ×300; (**C**) Hyaline membranes and intraalveolar extravasates, ×200; (**D**) Desquamation of bronchiolar epithelium, ×200. Hematoxylin-eosin staining.

Case 10. A 61-year-old male was admitted to the hospital on 23 October 2020, with a diagnosis of COVID-19 (U07.1). On October 24, the patient’s biological death was recorded from acute respiratory failure, which developed due to total bilateral polysegmental pneumonia with a hemorrhagic component caused by SARS-CoV-2. Histological examination of the Pt 10 lungs revealed signs characteristic mainly of the exudative phase of DAD: vascular congestion and capillary stasis (Figure 3A), intra-alveolar edema (Figure 3B), hyaline membranes (Figure 3B,C), intraalveolar extravasates, and cellular detritus (Figure 3C), and desquamation of the alveolar and bronchiolar epithelium (Figure 3D).

**Figure 4:**
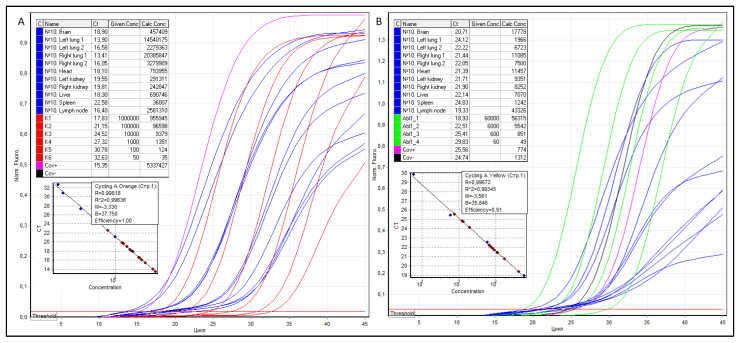
Threshold cycles (Ct), the concentration of serial dilutions of pGEM^®^-T Easy plasmids, the copy number of SARS-CoV-2 (**A**) and ABL1 (**B**) cDNA, as well as RT qPCR graphical curves of patient No. 10. The samples are colored blue, SARS-CoV-2 serial dilutions—red, ABL1 serial dilutions—green.

RNA was isolated from tissue sections of the brain, lungs, heart, kidneys, liver, spleen, and regional lymph node, cDNA was obtained by reverse transcription, and RT qPCR was performed, as shown in Figure 4.

According to the formula N^SARS-CoV−2^/N^ABL1^ × 100, the VL in the anterosuperior and posterior basal regions of the left and right lungs was 739,582, 33,904, and 183,905, 43,732 copies of SARS-CoV-2 cDNA per 100 copies of ABL1, respectively. Thus, the average VL in the lungs for four samples is 250,281 copies of SARS-CoV-2 cDNA per 100 copies of ABL1. The VL in the tracheobronchial lymph node, heart, left and right kidneys (mean), liver, spleen, and brain were 5958, 6930, 3115, and 2943 (3029), 9770, 2899, and 2573 copies of SARS–cDNA CoV-2/100 copies of ABL1, respectively.

**Figure 5:**
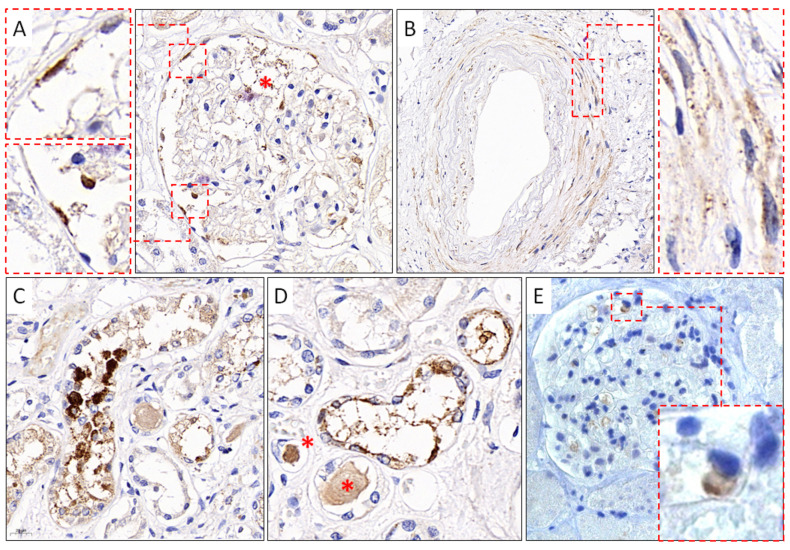
Results of IHC study using mAb against SARS-CoV-2 S-protein. (**A**). SARS-CoV-2–positive staining of endothelial cells, cells of the capillary network (red asterisk), and parietal epithelial cells (red frames) of the capsule of the renal glomeruli, GeneTex, ×400; (**B**). Immunoreactive smooth muscle cells (red frame) of the vascular wall of the kidneys, GeneTex, ×400; (**C**,**D**). Positive staining of cells of the convoluted tubules of the kidneys and homogeneous protein content in the lumen of the tubules (red asterisks), GeneTex, ×400; (**E**). Positive staining of the renal glomeruli capillary network cells (red frame), Abcam, ×400.

In addition to the results of IHC studies of the lungs, lymph nodes, and spleen, obtained by us earlier [50], in this work, we present the results of IHC studies of kidney tissue. Immunoreactivity to anti-S SARS-CoV-2 mAb (GeneTex) was detected in the endothelial cells of the capillary network and cells of the parietal epithelium of the renal glomerular capsule (Figure 5A), in the smooth muscle cells of the vascular wall of the kidneys (Figure 5B), and in the cells of the partially necrotic tubular epithelium of the kidneys (Figure 5C,D). In addition, when using polyclonal antibodies against the S-protein SARS-CoV-2 (Abcam), positive staining was observed in single cells of the capillary network of the renal glomeruli (Figure 5E).

**Figure 6:**
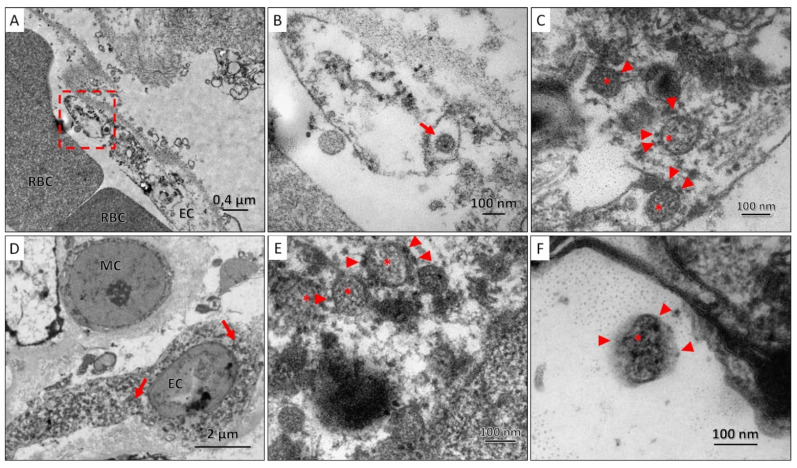
(**A**). Section of the air-blood barrier in which part of the endothelial cell (EC) and red blood cells (RBC) can be distinguished; SARS-CoV-2 virions were found in the cytoplasm of endothelial cells (red frame); (**B**). A coronavirus particle (red arrow) was found in the cytoplasm of the endothelial cell; (**C**). At high magnification, SARS-CoV-2 virions were characterized by a spherical shape, with an average size of 100 nm, surrounded by a membrane, on the surface of which there are electron-dense outgrowths of the S-protein (arrowhead), and granular nucleocapsid structures (asterisks); (**D**). A region of the vascular glomerulus of the kidney with mesangial (MC) and endothelial (EC) cells, in the cytoplasm of which SARS-CoV-2 virions are found closer to the nucleus (red arrows). (**E**,**F**). At high magnification, SARS-CoV-2 virions were characterized by a spherical shape, with an average size of 100 nm, surrounded by a membrane, on the surface of which there are electron-dense outgrowths of the S-protein (arrowhead), and granular nucleocapsid structures (asterisks) can be seen in the lumen of the particles.

TEM examination of lung and kidney tissues revealed coronavirus particles surrounded by a membrane with electron-dense outgrowths of the S-protein and granular structures of the nucleocapsid visualized in the lumen of the particles. SARS-CoV-2 virions were spherical, with an average size of 100 nm; they were found both individually and in the form of clusters enclosed in giant membrane intracellular vesicles with electron-lucent, almost transparent, homogeneous contents. Viral particles were found in the cytoplasm of the endothelial cell of the capillary closer to the periphery, and in the kidneys, they were also found in the cytoplasm of the endothelial cell, but predominantly perinuclear (Figure 6).

To confirm a possible link between SARS-CoV-2 infection and tissue damage to the heart, liver and, kidneys, we compared the SARS-CoV-2 VL levels in these tissues and the serum CPK, AST, ALT, LDH, ALP, and Cr levels, which are presented in Table 2.

As shown in Table 2, a comparison of the serum CPK and SARS-CoV-2 VL in the heart of COVID-19 patients showed no relationship between them. On the contrary, in patients with zero SARS-CoV-2 VL in the heart (No. 5–8, 19), the CPK level exceeded the expected value by 3–5 times (641–1007 IU/L), and in patients with high SARS-CoV-2 VL (No. 9, 23), the CPK level did not exceed the normal range. Furthermore, a significant increase in ALT and AST levels was characteristic for patients with uninfected liver tissue (No. 1, 7, 8, 11, 16) and patients with high SARS-CoV-2 VL in the liver (No. 4, 12, 1). The absence of any relationship was also found between the serum LDH, ALP, and Cr levels and SARS-CoV-2 VL in the examined organs of our patients. In addition, histological examination of the heart, liver, and kidney tissues with “zero” and high VL SARS-CoV-2 did not reveal any differences in the nature of pathological changes.

## 4. Discussion

Since the pandemic, a lot of data has been accumulated on the multisystem manifestations of COVID-19 infection, often caused by extrapulmonary dissemination and tropism of the SARS-CoV-2 to the tissue of vital organs, particularly to the heart, kidneys, liver, and brain of COVID-19 patients [16,17]. The detection rate of SARS-CoV-2 RNA in the lungs of our COVID-19 patients was 91.66% (33/36), which is comparable to the results of other studies where SARS-CoV-2 RNA was detected from 94 to 100% of cases [23,51]. The range of SARS-CoV-2 VL levels in the lungs of our COVID-19 patients ranged from 0 to 250,281 copies of SARS-CoV-2 DNA per 100 ABL1. At the same time, the average SARS-CoV-2 VL in the lungs with a predominance of histological features of the proliferative and exudative phases of DAD differed 60 times. Perhaps, therefore, the exudative phase of DAD of the lungs is considered the most favorable “temporal window” in which the probability of detecting SARS-CoV-2 in the lungs is the highest [52]. The mean time spent in the ICU for patients with mono-organ and multiple organ dissemination of SARS-CoV-2 was 15.8 and 10.8 days, respectively.

In our cohort of patients, the frequency of SARS-CoV-2 RNA detection in tracheobronchial lymph nodes was 58.8%, heart–40%, spleen–35.3%, liver–31.4%, kidney–20.7% and brain–9%. For comparison, the extrapulmonary dissemination of SARS-CoV-2 in the lymph nodes is 70% [40], heart–61.5%–82% [24,25], liver–55–82% [25,26], spleen–64.7% [25], kidney–58.8–77% [16,25], and brain–53–64.7% [25,28].

We assume that the high frequency of dissemination and the VL in the lymph nodes draining lymph from the lungs, the primary focus of the infection, indicate a possible lymphohematogenic spread of SARS-CoV-2 in conditions of impaired blood circulation. Our assumptions are confirmed by the results of other studies, where the highest SARS-CoV-2 VL was also detected in the hilar lymph nodes. However, the authors explained this phenomenon only with their topological proximity to the lungs [53].

The correlation between the higher SARS-CoV-2 VL and the exudative phase of DAD in the lungs, which we identified, was also noted by other authors [54,55]. In addition, we found a relationship between the VL level in the lungs and the time spent in the ICU before death (rS = −0.348, *p* = 0.037). According to this, patients with higher VL in the lungs had a shorter stay in the ICU, and vice versa.

A large number of studies have noted a correlation between the level of VL SARS-CoV-2 in swabs from the nasopharynx of patients at the onset of the disease and the risk of severe course and death of COVID-19 [56,57,58,59,60]. However, in our cohort of patients, SARS-CoV-2 VL in the lungs ranged from 0 to 250,281 copies of SARS-CoV-2 cDNA per 100 copies of ABL1, and no association was found between SARS-CoV-2 VL in the lungs and fatal outcome of COVID-19. We believe that the SARS-CoV-2 VL level in the primary focus of inflammation, in the lung tissue, and, moreover, in upper respiratory tract smears, cannot be an isolated prognostic factor for the risk of fatal outcome. The thanatogenesis of COVID-19 is the result of a combination of several factors, such as advanced age, the types and severity of comorbidities, and severe complications of COVID-19, including those associated with prolonged mechanical ventilation.

Isolated dissemination of SARS-CoV-2 into the heart muscle of patient №23 is not uncommon. For example, a similar case of arterial hypotension with cardiogenic shock or a case of death from myocarditis in a patient with isolated SARS-CoV-2 infection has been described by other researchers [45,59]. In addition, a series of cases of myocarditis with signs of cardiogenic shock in 7 young men aged 20 to 42 years with no risk factors for cardiovascular diseases indicates that myocardial injury is not uncommon in COVID-19 patients [60].

The results of IHC studies of lung tissue, lymph nodes, and spleen were presented in our previous work [50]. In addition, we revealed immunoreactivity to anti-SARS-CoV-2 mAb in endothelial cells of the capillary network and parietal epithelial cells of the renal glomerular capsule, in smooth muscle cells of the renal vessel wall, as well as in cells of the partially necrotic tubular epithelium of the kidneys. Similar results using similar antibodies were previously reported by other authors [31,61,62].

Our TEM study results are confirmed by the results of previously published works, revealing the presence of coronavirus virions mainly in vascular endothelial cells of the lungs [63], intestines [64,65], kidneys [66], heart [67], skin [68], brain [69], liver [70], pancreas [71]. Furthermore, the TEM data confirms the hypothesis about the critical role of endothelial dysfunction in the development of multiorgan failure and the fatal outcome of COVID-19 [72,73].

According to the literature, COVID-19-associated myocardial, hepatic and renal injuries are associated with elevated serum CPK, LDH, ALT, AST, ALP, and Cr levels [18]. However, when comparing the biomarker levels with the SARS-CoV-2 VL levels in the heart, liver, and kidneys, we identified no link between them. We assume that the pathogenesis of the heart, liver and kidney injuries, associated with the elevation of serum biomarkers of their functional activity (CPK, LDH, ALT, AST, ALP, and Cr), is due to a combination of several mechanisms, including cytokine storm [2,12], endothelial cell damage and vascular inflammation [14], and septic organ damage [15]. According to Chornenkyy et al., in the majority of patients who died from COVID-19 with high transaminase values and histological signs of hepatitis, coronavirus was detected in less than half of the cases [74]. Furthermore, according to Lagana et al., PCR positivity was not significantly associated with increased AST/ALT [26]. The lack of a relationship between the SARS-CoV-2 VL and the development of organ failure in COVID-19 patients has also been previously described by other authors [2,53]. In their opinion, the direct effect of the SARS-CoV-2 coronavirus on the tissue of vital organs is limited in time and is observed only at the onset of the disease. However, the inflammation process is supported by other mechanisms [2,75]. Histological study of SARS-CoV-2 infected tissues revealed only non-specific pathological changes, and the high VL levels were detected even in tissues with no histological signs of injury [25,53].

## 5. Conclusions

Thus, SARS-CoV-2 multi-organ dissemination was detected in 63.8% of patients who died from COVID-19. The “pulmonary factors” of SARS-CoV-2 multi-organ dissemination are high VL (≥4909 copies) and the exudative phase of DAD. The frequency of SARS-CoV-2 RNA detection in tracheobronchial lymph nodes was 58.8%, heart–40%, spleen–35.3%, liver–31.4%, kidney–20.7% and brain–9%. In conditions of impaired blood circulation, SARS-CoV-2 particles could be spread from the lungs via lymphohematogenic spread due to the high frequency of SARS-CoV-2 dissemination and the VL in the lymph nodes draining lymph from the lungs, the primary focus of the infection. The SARS-CoV-2 VL levels in the vital organ are not always associated with the serum level of CPK, LDH, AST, ALT, ALP, and Cr in patients who died from COVID-19. An isolated detection of SARS-CoV-2 RNA in the myocardium of a COVID-19 patient who died from heart failure is possible. The detection of SARS-CoV-2 virions within endothelial cells in various organs via TEM may confirm the leading role of vascular disorders in the pathogenesis of multiorgan failure.

## Figures and Tables

**Table 1:** General characteristics of patients and SARS-CoV-2 VL level by organs.

#	Sex	Age	Time ^1^	Phase of DAD ^2^/Other Pulmonary Findings	SARS-CoV-2 VL Level by Organs						
					Lungs ^3^	LN ^4^	Heart	Spleen	Liver	Kidneys ^5^	Brain
**Group 1: (*n* = 2) SARS-CoV-2 has not been detected**											
7	M	62	2	Proliferative	0	0	0	0	0	-	0
17	F	77	15	Proliferative/emphysema	0	0	0	0	0	0	0
**Group 2: (*n* = 11) SARS-CoV-2 mono-organ dissemination**											
23	M	90	9	Bronchopneumonia with fibrosis	0	0	1238	0	0	0	0
34	F	73	23	Bronchopneumonia	27	0	0	0	0	0	0
29	F	79	24	Bronchopneumonia	38	0	0	0	0	0	0
22	M	90	10	Proliferative	63	0	0	0	0	0	0
15	M	68	19	Bronchopneumonia	190	0	0	0	0	0	0
31	F	78	10	Proliferative	208	0	0	0	0	0	0
20	F	85	23	Bronchopneumonia	270	0	0	0	0	0	0
8	M	64	19	Proliferative	677	0	0	0	0	-	0
32	M	85	10	Proliferative	694	0	0	0	0	0	0
11	M	84	17	Proliferative	706	-	0	*n*/a	0	-	0
14	M	80	10	Proliferative	1952	0	-	0	-	-	0
**Group 3: (*n* = 23) SARS-CoV-2 multi-organ dissemination**											
5	F	73	18	Proliferative/bronchopneumonia with hemorrhages	18	0	0	0	70	0	0
6	M	66	27	Hemorrhages and fibrosis	313	0	0	0	7	0	0
33	F	86	3	Proliferative	810	96	0	0	0	0	0
13	F	71	1	Exudative	834	1318	974	629	602	-	718
35	F	73	2	Proliferative	910	215	197	0	0	0	0
21	M	67	13	Proliferative	982	180	0	0	0	0	0
30	M	61	5	Proliferative/bronchopneumonia	1002	237	0	0	0	0	0
1	M	52	1	Proliferative/emphysema	1016	112	0	0	0	0	0
25	M	74	22	Proliferative	1032	825	0	0	0	0	n/a
16	M	67	9	Proliferative/bronchopneumonia with hemorrhages	2657	1110	0	0	0	0	0
4	M	76	5	Exudative and early proliferative/hemorrhages	4909	195	1267	71	47	52	0
19	M	84	24	Exudative	7476	1807	0	0	0	0	0
9	F	72	12	Exudative/bronchopneumonia	12,116	3801	2223	-	0	0	0
28	F	95	7	Exudative	14,522	839	392	34	425	0	0
3	M	84	11	Exudative/bronchopneumonia with necrosis	14,937	162	3733	9	160	0	0
27	M	84	16	Exudative	16,867	1180	0	1320	522	0	n/a
18	M	85	16	Exudative	17,817	n/a	270	218	0	-	0
36	M	73	2	Exudative	18,219	2691	409	181	0	83	0
26	F	93	7	Exudative	27,349	1629	1222	213	256	878	0
2	F	86	25	Exudative	73,214	789	761	72	40	126	782
12	F	85	7	Exudative	151,183	513	538	1891	980	-	0
24	F	76	11	Exudative	159,217	11,586	2418	551	0	1291	n/a
10	M	61	6	Exudative	250,281	5958	6930	2899	9770	3029	2573

^1^ Number of days spent in ICU before the fatal outcome; ^2^ DAD, diffuse alveolar damage; ^3^ Mean VL level in four lung samples; ^4^ LN, lymph node; ^5^ Mean VL level in two kidney samples; “n/a”—the material was not available; “-”—no PCR amplification.

**Table 2:** SARS-CoV-2 VL levels in heart, liver, and kidney, and serum biomarker levels of 27 COVID-19 patients.

#	VL Heart	CPK, IU/L	VL Liver	AST, IU/L	ALT, IU/L	LDH, U/L	ALP, IU/L	VL Kidney	Cr, μmoles/L
NR		20–200		8–33	4–36	40–280	44–147		61.9–114.9
**Group 1. SARS-CoV-2 has not been detected**									
7	0	485	0	149	121	490	307	-	415
17	0	71.3	0	31.2	17.1	207.4	-	0	114.7
**Group 2. SARS-CoV-2 mono-organ dissemination **									
23	1238	154	0	46	33	323	192	0	194
22	0	-	0	25	11	395	189	0	309
15	0	-	0	26	26	555	162	0	103
20	0	-	0	62.2	42.5	-	-	0	185.3
8	0	775	0	72.5	87.35	836	-	0	96.41
11	0	83	0	978	503	2058	239	0	82
14	0	-	-	58	41	564	-	0	109
**Group 3. SARS-CoV-2 multi-organ dissemination**									
5	0	1007	70	93	35	-	337	0	228
6	0	656.6	7	33.2	42.6	396.7	-	0	98.7
13	974	290.8	0	47.1	20.5	358.2	-	-	83.6
21	0	197	0	73.9	58.7	637.9	-	0	129.7
1	0	-	0	813	390	-	-	0	296.1
25	0	145.4	0	31	31.7	649.5	-	0	156.2
16	0	-	0	170.5	56.1	-	-	0	104.6
4	1267	722	47	73	73	626	-	52	194
19	0	641.4	0	140.8	81.7	493.3	-	0	283
9	2223	29	0	18	14	-	-	0	75.2
3	3733	-	160	75	-	-	-	0	122
27	0	-	522	35	22	-	-	0	216
18	270	260	0	23	21	289	48	-	89.6
26	1222	495	256	43	18	428	206	878	353
2	761	1327	40	54	13	-	-	126	364.4
12	538	735.4	980	594.6	786.6	902.7	-	-	193.6
24	2418	-	0	16,3	8.6	-	-	1291	137.4
10	6930	609	9770	71	62	767	-	3029	199

Note: CPK, creatine phosphokinase; AST, aspartate aminotransferase; ALT, alanine aminotransferase; LDH, lactate dehydrogenase; ALP, alkaline phosphatase; Cr, creatinine; NR, normal range; elevated serum biomarkers levels were colored red; “-” the data not available.

## Data Availability

The data presented in this study are available on request from the corresponding author.
